# User and Developer Views on Using AI Technologies to Facilitate the Early Detection of Skin Cancers in Primary Care Settings: Qualitative Semistructured Interview Study

**DOI:** 10.2196/60653

**Published:** 2025-01-28

**Authors:** Owain Tudor Jones, Natalia Calanzani, Suzanne E Scott, Rubeta N Matin, Jon Emery, Fiona M Walter

**Affiliations:** 1 Department of Public Health and Primary Care University of Cambridge Cambridge United Kingdom; 2 Institute of Applied Health Sciences University of Aberdeen Aberdeen United Kingdom; 3 Wolfson Institute of Population Health Queen Mary University of London London United Kingdom; 4 Department of Dermatology Churchill Hospital Oxford United Kingdom; 5 Centre for Cancer Research, and Department of General Practice and Primary Care University of Melbourne Melbourne Australia

**Keywords:** artificial intelligence, AI, machine learning, ML, primary care, skin cancer, melanoma, qualitative research, mobile phone

## Abstract

**Background:**

Skin cancers, including melanoma and keratinocyte cancers, are among the most common cancers worldwide, and their incidence is rising in most populations. Earlier detection of skin cancer leads to better outcomes for patients. Artificial intelligence (AI) technologies have been applied to skin cancer diagnosis, but many technologies lack clinical evidence and/or the appropriate regulatory approvals. There are few qualitative studies examining the views of relevant stakeholders or evidence about the implementation and positioning of AI technologies in the skin cancer diagnostic pathway.

**Objective:**

This study aimed to understand the views of several stakeholder groups on the use of AI technologies to facilitate the early diagnosis of skin cancer, including patients, members of the public, general practitioners, primary care nurse practitioners, dermatologists, and AI researchers.

**Methods:**

This was a qualitative, semistructured interview study with 29 stakeholders. Participants were purposively sampled based on age, sex, and geographical location. We conducted the interviews via Zoom between September 2022 and May 2023. Transcribed recordings were analyzed using thematic framework analysis. The framework for the Nonadoption, Abandonment, and Challenges to Scale-Up, Spread, and Sustainability was used to guide the analysis to help understand the complexity of implementing diagnostic technologies in clinical settings.

**Results:**

Major themes were “the position of AI in the skin cancer diagnostic pathway” and “the aim of the AI technology”; cross-cutting themes included trust, usability and acceptability, generalizability, evaluation and regulation, implementation, and long-term use. There was no clear consensus on where AI should be placed along the skin cancer diagnostic pathway, but most participants saw the technology in the hands of either patients or primary care practitioners. Participants were concerned about the quality of the data used to develop and test AI technologies and the impact this could have on their accuracy in clinical use with patients from a range of demographics and the risk of missing skin cancers. Ease of use and not increasing the workload of already strained health care services were important considerations for participants. Health care professionals and AI researchers reported a lack of established methods of evaluating and regulating AI technologies.

**Conclusions:**

This study is one of the first to examine the views of a wide range of stakeholders on the use of AI technologies to facilitate early diagnosis of skin cancer. The optimal approach and position in the diagnostic pathway for these technologies have not yet been determined. AI technologies need to be developed and implemented carefully and thoughtfully, with attention paid to the quality and representativeness of the data used for development, to achieve their potential.

## Introduction

### Background

Skin cancers are among the most common cancers worldwide, with increasing incidence in most populations [1,2]. Melanoma is the most lethal skin cancer, but keratinocyte cancers (KCs), which include squamous cell carcinomas and basal cell carcinomas, comprise most skin cancers [1-3]. There were >200,000 skin cancer diagnoses in England in 2021, comprising 193,000 nonmelanoma skin cancers (which include KCs) and nearly 16,000 melanomas [4]. The World Health Organization estimates that 2 to 3 million nonmelanoma skin cancers and 132,000 melanomas occur globally each year [5]. Earlier diagnosis of skin cancers is associated with statistically significantly better outcomes [3]. In the United States, early detection of melanoma is associated with >99% five-year survival but falls to 74% when it has spread to lymph nodes and 35% when spread to distant organs [6].

There has been a substantial interest in applying artificial intelligence (AI) to the diagnosis of skin cancer through visual analysis of skin lesions, either through a smartphone app or uploading images of skin lesions. Many AI technologies have been designed for use by patients and a handful for use by clinicians as clinical decision aids [7,8]. Most of these technologies do not have the appropriate regulatory approvals in place to support their safety and efficacy when used in these settings [9], with limited evidence on their efficacy and accuracy from clinical trials or real-life clinical settings [7,8], although some evidence is emerging [10,11]. There is limited evidence on how users in clinical settings interact with AI technologies and how this might affect patient safety [12], and a lack of qualitative studies reporting public perspectives [13].

**Figure 1:**
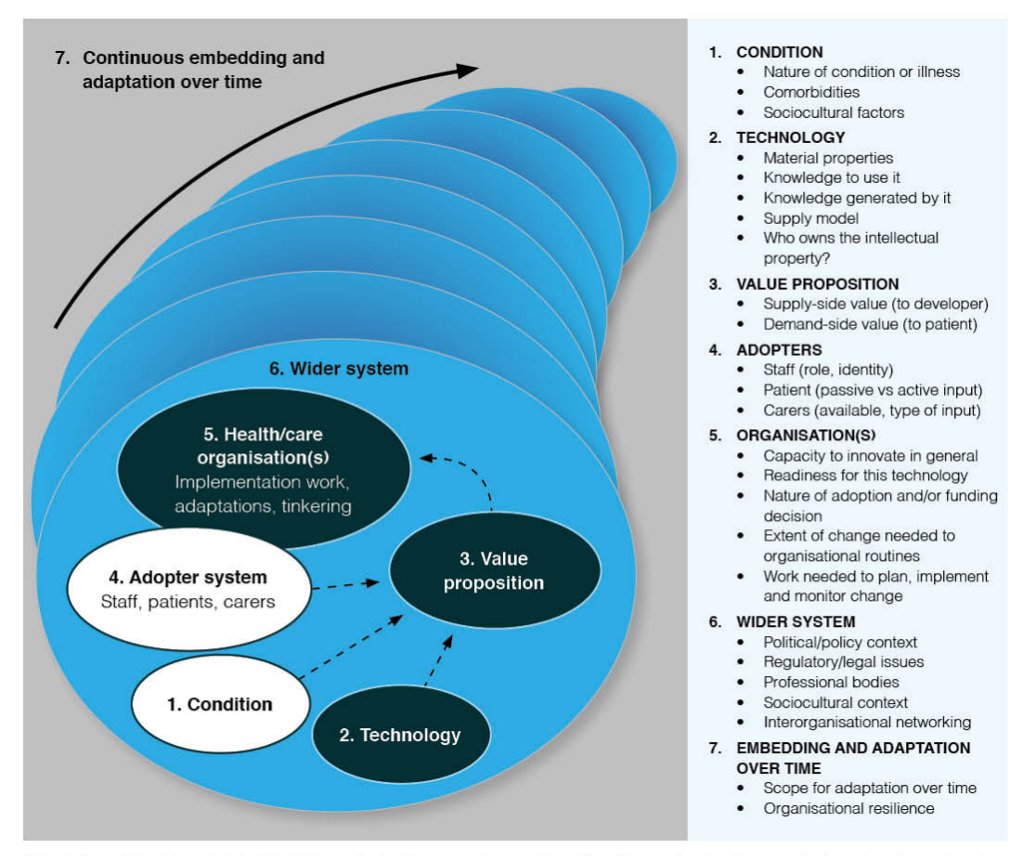
The Nonadoption, Abandonment, and Challenges to Scale-Up, Spread, and Sustainability (NASSS) framework [15].

Implementation of a diagnostic technology in clinical settings is a complex process and prone to failure—a technology must pass through several stages of development before implementation is likely to be successful [14]. Several frameworks analyze factors around the implementation of new technologies. In this study we chose to use the Nonadoption, Abandonment, and Challenges to Scale-Up, Spread, and Sustainability (NASSS) framework [15] to help understand and interpret the data. This framework incorporates complexity principles and allows researchers to identify and explain the manifestations of complexity in technology-supported change projects (Figure 1) [15]. We believe that these attributes made it best suited to this study compared with other conceptual frameworks for implementation research.

### Objectives

The aim of this study was to consult several stakeholder groups (ie, groups that may have views or concerns about the use of AI to help diagnose skin cancer in primary care) to provide in-depth understanding of barriers and facilitators to the implementation of AI technologies for the early diagnosis of skin cancers in primary care settings. Depending on how AI is implemented in the skin cancer diagnostic pathway, users of the technology could include members of the public, patients, general practitioners (GPs), primary care nurse practitioners (NPs), and dermatologists. Dermatologists also receive most referrals from primary care for suspected skin cancers. AI researchers working in both academic and commercial settings are the primary developers of AI technologies. The views of all these groups were important to understand.

## Methods

### Design

This was a qualitative study that was performed using semistructured interviews of stakeholders.

### Recruitment and Sampling

Four groups were selected for the study: (1) members of the public, (2) patients previously diagnosed with skin cancer, (3) health care professionals (HCPs; including GPs, primary care NPs, and dermatologists), and (4) AI researchers from academic and commercial settings. Members of the public were approached via the Cambridge Biomedical Research Centre Patient and Public Involvement (PPI) group and “snowballing” invitations to their colleagues. Members of the public with a history of skin cancer were included in the patient group, with additional patients approached via Melanoma Focus (a UK melanoma charity that provides information, guidance, and support for patients, carers, and HCPs) [16]. GPs and primary care NPs were identified through the Primary Care Dermatology Society [17], with additional GPs approached via Sermo (medical market-research organization) [18]. Dermatologists were identified from the British Association of Dermatologists (BAD) [19] and through snowballing. AI researchers from academic settings were identified through contacts within academic institutions, and AI researchers from commercial settings were recruited via email from companies identified in 2 reviews [7,8]. In this paper, we use primary care practitioner (PCP) to denote any medical practitioner that works in a primary care setting and might consult with a patient about a suspicious skin lesion, including GPs, family doctors, NPs, physician assistants, and paramedic practitioners. HCP is used when we refer to the views of wider HCPs, including secondary care HCPs. Staff who work in primary and community care and do not have clinical training and experience in the diagnosis of skin cancer but could potentially use an AI technology with patients with suspicious skin lesions are referred to as allied HCPs; a wide variety of professions could be included in this group, but it certainly includes practice nurses, health care assistants, clinical navigators, pharmacists, and podiatrists.

Participants were sampled to achieve a spread of age, sex, and geographical location within each participant group. Patients and the public were recruited to achieve a spread of ages >60 years and <60 years, reflecting the average age of skin cancer diagnosis. HCPs and AI researchers were recruited to achieve a spread of ages >45 years and <45 years, reflecting the midcareer point of these professions. Patients were sampled to include a range of prior history of skin cancer types. GPs were sampled to include a spread of roles (GP partner, salaried GP, locum GP, GP with extended role in dermatology). Participants were asked how supportive they were of using AI technologies to help diagnose skin cancer in primary care (using a Likert scale of strongly disapprove, disapprove, neutral, approve, and strongly approve). We aimed to recruit at least 1 participant in each group who disapproved and at least 1 who approved of the use of AI in this setting to obtain a range of perspectives.

### Ethical Considerations

Ethics approval for this study was granted by the Cambridge Psychology Research Ethics Committee (PRE.2021.098). Participants gave informed written and verbal consent to take part in the interviews. Patient and public participants were invited to bring a friend or family member with them to take part in the study if they wished. All interviews were digitally recorded and transcribed verbatim by a professional transcription company. Transcripts were checked and anonymized before analysis. To facilitate recruitment, a £20 (US $25.24) Apple iTunes or Google Play voucher was offered to all participants.

### Data Collection

An interview topic guide was developed with input from our PPI group to explore views on facilitators and barriers to the use of AI technologies to help diagnose skin cancer in primary care. All interviews were conducted by OTJ, who has a clinical background, with guidance from NC, a health services researcher with expertise in qualitative research. Interviews took place on the web using Zoom (Zoom Video Communications) at a time of the participants choosing between September 9, 2022, and May 25, 2023. Interview schedules for patients and members of the public and HCPs and AI researchers are available in Multimedia Appendix 2. Details on the interviews are reported in the COREQ (Consolidated Criteria for Reporting Qualitative Research; Multimedia Appendix 3) checklist. Interviews continued until we had a rich, multifaceted dataset. A reflexivity journal with field notes was kept and was discussed at regular meetings during the study.

### Analysis

Interviews were analyzed inductively and deductively using thematic framework analysis [20]. Two researchers (OTJ and NC) repeatedly read the first 5 transcripts to become familiar with the data and generate initial codes. These initial codes were compared with the NASSS framework (Figure 1) [15] to generate a comprehensive list of codes. The remaining transcripts were then read and indexed using NVivo (version 14; Lumivero) [21], with codes modified or created as required where the data did not fit comfortably into the NASSS framework. Coding was completed by OTJ with a sample of transcripts (7/29, 24%) checked by NC. Codes were defined and discussed regularly in team meetings (OTJ, NC, and FMW), and coding files were saved throughout the process to maintain an audit trail of changes to the code tree. Data were charted into Microsoft Excel [22], and the characteristics, similarities, and differences between data were identified. Relationships and connections between categories were then mapped to generate the themes presented in the results. Themes were further refined with guidance from senior team members (FMW and SS [health psychologist]). Together this broad authorship group of clinical academics and behavioral scientists added rigor to data analysis and interpretation.

## Results

### Participants

A total of 29 interviews were conducted with members of the public (n=6, 21%), patients (n=5, 17%), HCPs (n=13, 45%), and AI researchers (n=5, 17%; Table 1). Participants were recruited via mailing lists and social media; therefore, the denominator is unknown.

**Table 1:** Participant demographics.

		Overall (n=29), n (%)	Public^a^ (n=6), n (%)	Patients^b^ (n=5), n (%)	HCPs^c^ (n=13), n (%)	AI^d^ researchers (n=5), n (%)
**Sex**						
	Male	10 (34)	1 (17)	1 (20)	4 (31)	4 (80)
	Female	19 (66)	5 (83)	4 (80)	9 (69)	1 (20)
**Age group^e^ (y)**						
	>60	N/A^f^	2 (33)	2 (40)	N/A	N/A
	<60	N/A	4 (67)	3 (60)	N/A	N/A
	>45	N/A	N/A	N/A	7 (54)	1 (20)
	<45	N/A	N/A	N/A	6 (46)	4 (80)
**Location**						
	England	23 (79)	5 (83)	5 (100)	11 (85)	2 (40)
	Wales	2 (7)	1 (17)	0	1 (8)	0
	Scotland	1 (3)	0	0	1 (8)	0
	Outside United Kingdom^g^	3 (10)	0	0	0	3 (60)
**Support for using AI to facilitate the early diagnosis of skin cancers in primary care**						
	Strongly approve	10 (34)	2 (33)	1 (20)	5 (38)	2 (40)
	Approve	15 (52)	3 (50)	3 (60)	6 (46)	3 (60)
	Neutral	2 (7)	0	1 (20)	1 (8)	0
	Disapprove	1 (3)	1 (17)	0	0	0
	Missing data	1 (3)	0	0	1 (8)	0
**Highest educational qualification^h^**						
	Foundation or intermediate qualifications	0 (0)	0	0	0	0
	Advanced qualifications	1 (3)	0	1 (20)	0	0
	Higher qualifications	28 (97)	6 (100)	4 (80)	13 (100)	5 (100)
**Ethnicity**						
	Black African	1 (3)	1 (17)	0	0	0
	Middle Eastern	1 (3)	0	0	0	1 (20)
	Missing data	3 (10)	0	1 (20)	1 (7)	1 (20)
	White British	18 (62)	5 (83)	3 (60)	10 (77)	0
	White European	6 (21)	0	1 (20)	2 (15)	3 (60)
**History of skin cancer**						
	Yes	N/A	0	5^i^ (100)	N/A	N/A
	No	N/A	6 (100)	0	N/A	N/A
	Skin cancer in a family member or close friend	N/A	2 (33)	5 (100)	N/A	N/A

^a^Occupations: project manager, lawyer, pharmaceutical industry, music industry, biomedical scientist, or missing data (n=1 for each).

^b^Occupations: fraud services, social researcher, teacher, accountant, and research consultant (n=1 for each).

^c^HCP: Health care professional. HCPs included general practitioners (GPs, n=6), primary care nurse practitioners (n=3), and dermatologists (n=4).

^d^AI: artificial intelligence.

^e^Patients and the public were recruited to achieve a spread of participants aged >60 and <60 years. HCPs and AI researchers were recruited to achieve a spread of ages >45 and <45 years.

^f^N/A: not applicable.

^g^Including the Netherlands and North America.

^h^Educational qualification categories were taken from the UK National Census 2021. Definitions are available on the internet [23].

^i^Basal cell carcinoma (n=3), melanoma (n=1), and Merkel cell carcinoma (n=1).

**Figure 2:**
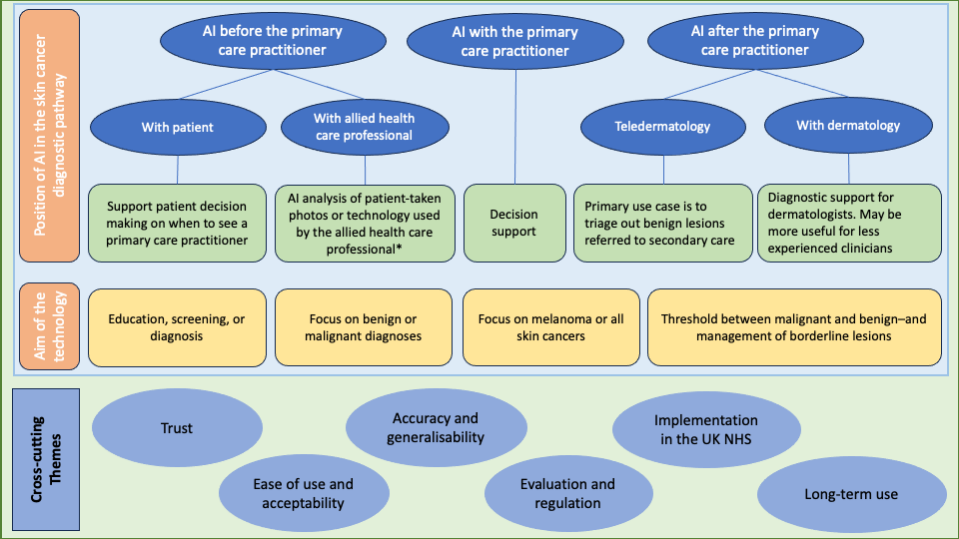
Issues around the design and intended positioning of artificial intelligence (AI) technologies for the diagnosis of skin cancer that were identified from the data, and further cross-cutting themes identified. *Allied health care professionals that could use the technology include pharmacists, health care assistants, practice nurses, podiatrists, hairdressers, and potentially many more. UK NHS: UK National Health Service.

The *Results* section is structured around the major themes and subthemes generated from the data. Major themes included the “position of AI in the skin cancer diagnostic pathway” and the “aim of the AI technology.” There were several cross-cutting themes, including trust, acceptability, generalizability, evaluation, regulation, implementation, and long-term use (Figure 2). Participant quotations are identified by participant group, sex, and age.

### Position of AI in the Skin Cancer Diagnostic Pathway

#### AI Before the PCP

Patients and members of the public felt that giving patients access to AI technology would be more accurate than “random googling” and could help them to decide when to see a physician. However, they expressed concerns about using the AI technology without clinical input. The loss of the human touch in consultations worried them, including how the diagnosis would be communicated and whether patients would be able to get a PCP follow-up appointment to discuss the diagnosis and ask questions. Another concern was whether all patients would be able to effectively use the technology and the potential for it to exclude some groups of patients, including older people. Patients and the public discussed the risk of overuse of the technology by patients; they felt that implementing it in a practice nurse and allied HCP-led clinic would reduce the risk of overuse while also making it easier for patients to access skin lesion assessments.

All HCPs could envision AI technology being used by patients and thought there would be a high demand for this service. Some dermatologists felt this could be a useful approach, particularly for patients at high risk of skin cancer between dermatology clinic appointments; however, they were concerned about the potential for overuse and subsequent workload due to false positives that would inevitably occur. They thought patients might struggle to understand the risks, benefits, and the output of the AI technology and would find it difficult to take high-quality images needed for the AI to analyze. Some HCPs commented on the potential psychological benefits of AI technology, in particular reducing patient anxiety if the technology reduced the number of urgent suspected cancer referrals, although some HCPs felt that it could increase patient anxiety through increasing access to skin lesion checks. Dermatologists and NPs commented on how accurate AI technology could enable a wide variety of allied HCPs to use the technology to triage patients presenting with suspicious skin lesions, which may improve access and enable earlier detection of skin cancer. GPs reported both positive and negative views on the potential of positioning AI technologies in the role of triaging patient-submitted photographs before an appointment with a PCP.

AI researchers from academic backgrounds were broadly skeptical of patients having access to AI technology themselves, highlighting concerns about the diagnostic accuracy of the technology, the risk of false reassurance, and that patients would not necessarily understand the context of when it was safe to use. However, AI researchers from commercial backgrounds felt that, if the AI technology was accurate enough, positioning AI technologies with patients had the greatest potential for impact. They highlighted various potential positive effects, including reducing the barriers to getting a skin lesion assessed, helping patients make better decisions about when they needed to see an HCP, and educating patients about skin cancer.

#### AI With the PCP

Patients commented that GP surgeries are accessible for most patients and so are an ideal place to locate the technology. They felt that if they were consulting about a potentially serious skin cancer (ie, melanoma), they would like to be able to speak to a PCP. Combining the clinical judgment of the PCP with the AI technology was generally viewed as being more powerful than the PCP alone and could be more beneficial for PCPs with less experience in skin cancer and reduce the variability in skin cancer triage in primary care. The public discussed whether PCPs would become reliant on the AI technology and lose their clinical judgment and even whether this was an issue if the AI diagnostic accuracy were better than the PCP’s.

GPs largely wanted PCPs to have access to the AI technology and were concerned that the implementation of AI technology at other points in the diagnostic pathway could undermine their gatekeeping role. They believed that PCPs are well positioned to triage and monitor skin lesions over time and provide continuity of care and that it would be more efficient for the technology to be used to triage a lesion in a single consultation instead of needing a separate appointment to capture the image. NPs agreed that AI technology would be best placed with a PCP. One GP emphasized that the positioning of the technology in the diagnostic pathway is probably the most important factor in how impactful it will be.

Dermatologists expressed the opinion that something was needed to improve the accuracy of referrals to secondary care but were not sure implementing AI technology in primary care was the best approach. A broad educational program for PCPs was suggested as a better alternative. Whether AI technology is currently accurate enough to be used by PCPs was a concern, as well as the risk of deskilling PCPs in skin lesion recognition. Dermatologists did see benefits in situating AI technology with PCPs to help with rarer skin cancer recognition, to break bad news, and answer patient questions, and to give PCPs more confidence in diagnosing a lesion as benign and not referring on to secondary care.

Most AI researchers expressed strongly that a “human-in-the-loop” approach (ie, where a human clinician is always involved in decision-making alongside the AI) is needed. Potential benefits of this approach included increased safety, allowing for patient interactions, allowing the PCP to focus more on the patient, and increasing knowledge of PCPs. AI researchers also discussed that the decision to biopsy or refer a skin lesion involves many biopsychosocial and clinical factors outside of the appearance of the lesion, which cannot be measured or considered by the AI and thus needs input from a PCP.

#### AI After the PCP

Use of AI technology to triage referrals from primary care to secondary care in a teledermatology setting was only mentioned by 1 GP and 2 dermatologists. The dermatologists suggested AI technology could help triage obviously benign skin lesions, preventing them from being referred to a dermatology clinic.

Few participants commented on positioning an AI technology with dermatologists. Dermatologists believed that it could be a useful training tool, and in the future, if it were proven to be more accurate than dermatologists, then it could be implemented in dermatology clinics. One AI researcher agreed and discussed how AI technology could help improve dermatologists’ consistency in diagnosis (Textbox 1).

**Positioning of artificial intelligence (AI) in the skin cancer diagnostic pathway.**

**AI before the primary care clinician**

- So I think people first self-diagnose via Google a lot anyway so having something that’s a little bit more accurate than self-diagnosis is probably helpful. [Patient 4; female, aged <60 years]
- I don’t think it would be effective giving it directly to patients, least of all because they won’t have the training and the right level of professional knowledge in being able to interact with digital innovations effectively. [Patient 1; male, aged <60 years]
- I would be a little bit concerned about it just being open-ended, because...the potential for over-use by some people and under-use by others would be quite large, I think. So, I think...a nurse-led clinic or something like that might be better. Or give people the option. [Member of the public 3; female, aged >60 years]
- I know from doing online and video consultations during COVID, what was quite evident is that patients have got varying skills in terms of how they use their mobile devices to show you their skin remotely and taking images, and the quality of those images. So I think you’d have to think very carefully about who is taking the image, what they understand about what the clarity of that image needs to be, and what devices they’re being used on. Because that’s going to affect your interpretation. So I would be hesitant to say as a matter of routine for it to be patient led. [NP1 (nurse practitioner 1); female, aged >45 years]
- And seeing this opportunity, for example, hairdressers, podiatrist, pharmacist, they get asked a lot about these things, about lesions. And from time to time you get referrals because they went to the hairdresser and they spotted a mole... it would be a good thing to implement in these areas, expand beyond the GPs. [Dermatologist 3; female, aged <45 years]
- I would really like to then streamline the process. So we have an ability within our practice that the patient doesn’t need an appointment for asking about a mole of concern and they can send a photo. That photo would be reviewed by a clinician. It would be absolutely fantastic if that photo was also able to be reviewed by an AI process and advise whether there were concerning features on it or not. It would be useful if that photo could then be reviewed again six weeks later against a new photo to assess for changes. [GP5 (general practitioner 5); male, aged <45 years]
- Yeah, it (using the app) has especially low barrier to use it so it’s way lower than going to a doctor or going to a dermatologist. It is really a first step into being more interested or more concerned about skin cancer. And I think it can help to raise awareness (of skin cancer) and inform people better. [AIR4 (AI researcher 4); male, aged >45 years]

**AI with the primary care clinician**

- I don’t want to think that a doctor would see results on an app and still not think about it at all...But I think with time they might lose their skills. [Member of the public 6; female, aged <60 years]
- I’d love it in my hands. Anything that saves primary care queuing people up in dermatology...I think skin is difficult. And so my feeling is that human error will always exist and I guess if you’ve got something to support you in making the correct diagnosis in serious skin lesions, then it seems to me like a win-win and a sensible option. [GP2; male, aged >45 years]
- Most patients with a lesion will go straight to their GP. So I think definitely that’s where it’s going to be best placed and whether that’s a specialist nurse within a GP setting or a GP, certainly within that primary care set up I think is most relevant. [NP1 (nurse practitioner 1); female, aged >45 years]
- I think selfishly, I’d be disappointed if I was told you don’t need your dermatoscope anymore, because it’s an area I’m really interested in and I’m really enjoying...because if it’s automated and, therefore, you lose the skill set and you lose motivation around the topic. [NP3; male, aged <45 years]
- I would be worried about de-skilling our GPs by giving them a tool that tells them what to do. [Dermatologist 3; female, aged <45 years]
- The great thing would be if they were able to pick up those skin cancers that they haven’t thought about, for example, amelanotic melanomas, or nodular melanomas, that they don’t follow the typical a, b, c, d criteria. [Dermatologist 3; female, aged <45 years]
- If AI had a really, really good dataset of benign lesions, that would give a GP confidence to say ‘no, that’s benign, that doesn’t need to be referred in’ [Dermatologist 4; female, aged >45 years]
- They (dermatologists) are very experienced and they don’t need a tool. But some of those tools might be needed for the people who refer patients, not to make any mistake at that stage for the early detection...So I can think of those two aspects, like speed and a better decision for the practitioner. I see really big opportunities in those kinds of things. [AIR2; male, aged <45 years]

**AI after the primary care clinician**

- So,...either a patient-generated image, but preferably taken in primary care on a high-quality camera and sent securely to dermatology for triage, then the AI helping with that triage process. [GP3; male, aged <45 years]
- In secondary care it would be really helpful, I think particularly for juniors starting out, to have a list of the differential, including the rarer things that could possibly be consistent with the appearances that the algorithm has identified. But giving you ranking of likelihood. [Dermatologist 2; female, aged >45 years]

### Aim of the Technology

#### Education, Screening, or Diagnosis

Participants mainly discussed AI technology as a diagnostic tool; however, participants from all groups raised alternative uses. Patients discussed skin self-monitoring, including sequential monitoring of skin lesions over time, and patient education about the “red flags” of skin cancer. All groups discussed the potential to raise awareness and educate patients about skin cancer, including skin cancer prevention and how to perform skin self-monitoring.

GPs and NPs discussed the potential for AI technologies to educate PCPs and improve diagnostic skills. They suggested that a potent educational attribute would be if the AI technology could highlight visual features of skin cancer in images of skin lesions; AI researchers commented that these types of features have been developed. Dermatologists discussed the potential use of a patient-facing AI technology as a screening tool for high-risk populations.

#### Focus on Benign or Malignant Diagnoses

Patients and members of the public stated that the primary aim of the AI technology should be a very high accuracy to avoid missing skin cancers and giving false reassurance; to achieve this, the technology would need to have a high sensitivity. All HCP groups commented that aiming to diagnose benign skin lesions might be a safer approach with less risk of missing skin cancers. They proposed that focusing on diagnosing specific common benign skin lesions, such as seborrheic keratoses and dermatofibromas might reduce unnecessary referrals to secondary care.

#### Focus on Melanoma or All Skin Cancers

Patients were primarily concerned that any AI technology was accurate for melanoma. Some members of the public had experience of KCs in family or friends and felt AI technologies should also address KCs. GPs were primarily concerned about melanoma because it often affects younger patients and has higher mortality—they felt that AI technologies could be applied to other skin cancers in the longer term. Dermatologists and a NP commented that diagnosing all types of skin lesions, including all skin cancers, was important, especially for technologies that are designed to be used by patients or HCPs with less experience in diagnosing skin cancer. However, some dermatologists thought it could be difficult to train AI to diagnose KCs accurately, because patient history often has greater importance than for melanoma, and AI currently does not always incorporate this.

#### Threshold Between Malignant and Benign and Management of Borderline Lesions

All groups discussed the difficulty in setting a threshold between benign and malignant lesions for the AI technology, in essence how to translate the continuous risk score generated by the AI into a binary clinical decision of whether to refer a patient or biopsy a skin lesion. A 3-layer management strategy was suggested by several participants with further assessment of lesions that are close to the threshold, either through follow-up assessment in primary or secondary care or through sequential monitoring over time (similar to short-term sequential digital dermoscopy imaging models that already exist [24]). NPs and dermatologists reflected on how this issue demonstrates the complexity of clinical practice and that clear guidelines will be needed about what to do at each risk level (Textbox 2).

**Aim of the artificial intelligence (AI) technology.**

**Education, screening, or diagnosis**

- I guess you always take a picture at a point in time and...say you noticed it change in a couple of months could you go back and say, it’s changed from this to this, and almost keeping a progression record...I think that would be a really helpful feature. [Patient 4; female, aged <60 years]
- Give people recommendations of you should not go out unless you’re wearing 50 SPF, we think you should not sunbathe between 12 and four, just because it’s cloudy does not mean you’re not going to burn...put a kid in a sun hat so they don’t have sunstroke. [Member of the public 3; female, aged >60 years]
- I say to them (patients) there are mole apps that you can monitor, and that maybe it’s not great in terms of getting a diagnosis, but they’re more aware and they’re keeping attention more to a particular lesion. And definitely it helps to diagnose cancer in the early state than what we saw years ago. [GP1 (general practitioner 1); female, aged <45 years]
- But the way I used AI was to try to train on some features in the image rather than giving me a diagnosis. For instance, to teach a deep learning algorithm to tell me whether that the borders are regular or irregular, ’cause that’s something subjective sometimes between readers...So I used AI to reveal the features rather than giving the full diagnosis. [AIR2 (AI researcher 2); male, aged <45 years]
- So if you could develop one that was validated it might be useful for selected patients, maybe not the entire population but particularly high risk patients, maybe patients with lots of moles. [Dermatologist 2; female, aged >45 years]

**Focus on benign or malignant diagnoses**

- The first aim of the app needs to be to go and get it checked or not, or to go and get it biopsied or not. [Patient 4; female, aged <60 years]
- Just screening out the seborrheic keratosis, the pigmented dermatofibromas, the benign...if it could screen all of those out, which are the vast majority of the two-week-wait referrals that we see, that would be incredibly important for providing a better, more targeted service...plus potentially triaging things that are so unlikely to be a cancer that they don’t need to be referred. So it kind of works both ways, early detection and reducing the massive numbers of 2-week-wait referrals that we get. [Dermatologist 2; female, aged >45 years]

**Focus on melanoma or all skin cancers**

- I think melanoma is the most important because I’d like to think that because your differential features for a squamous cell are quite obvious, that would be referred on anyway. I think it’s melanoma and other pigmented lesion differentiation that’s really tricky. So my thought would be more melanoma, and certainly that’s what I’ve used it for. [NP1 (nurse practitioner 1); female, aged >45 years]
- People just think it’s about diagnosing melanoma, where actually it’s not. It’s about recognising all skin lesions. You’ve got to diagnose benign lesions, and you’ve to diagnose malignant lesions, and it’s not just melanomas, you’ve got BCCs, you’ve got SCCs, you’ve got AKs, and then you’ve got all the benign lesions. [Dermatologist 1; female, aged >45 years]

**Threshold between malignant and benign—and management of borderline lesions**

- I guess one approach you could take with marginal cases, you could say that we suggest you get re-tested in six months’ time or something like that. So, you have a three-layer band. One you definitely need action, one you definitely don’t, and then a middle level where you come back for a test after a bit. [Member of the public 3; female, aged >60 years]
- What I suggest would be good...if you’re less than 70 per cent sure, it’s an arbitrary...whatever number sure, then there is clinician involvement. So take a deeper dive into the history and that person then comes in and is looked at. [NP3; male, aged <45 years]
- So I think it would be really interesting, if when I looked at a lesion I gave the patient a percentage of how right I thought I was or even any diagnosis. I mean, I’d love that, that would be really cool to have that honesty. ‘I think you’ve got a viral chest infection, I’m about 50-50, I’m going to hold off on the antibiotics, but here’s some strict safety netting.’ Yeah, I don’t know whether I’d want to give the patient that information. [NP3 male, aged <45 years]
- So it would come back with a comment like ‘this is 80% likely to be a melanoma, this is 20% likely to be a melanoma’ and there would have to be some sort of understanding, some sort of cut off, what is the point at which a referral is merited...And I suppose that one of the dangers...where do you cast the net in terms of risk? [Dermatologist 4; female, aged >45 years]

### Cross-Cutting Themes

#### Trust

Patients and members of the public often raised the issue of trust. The newness of the technology, the involvement of private companies and concerns about data privacy, and the diagnostic accuracy being <100% were all felt to be reasons for a lack of trust. However, there were also participants who thought patients would trust a consultation with a PCP more if it involved AI technology, even if it had made no difference to the assessment.

GPs worried that patients could demand the AI technology be used in consultations and were concerned about the risk of false reassurance. NPs felt that patients fundamentally trust people more than machines. Dermatologists stated that there is not enough evidence that existing AI technologies are safe and accurate enough to be used in clinical practice and that it is difficult for patients to determine which patient-facing AI technologies they can trust.

AI researchers discussed that the “black box” nature of AI technologies should not be a barrier to trust, as we similarly do not understand the mechanism of action of many medications. They felt that if clinicians recommended or adopted an AI technology, then patients would be more likely to trust it.

#### Ease of Use and Acceptability

Ease of use was a major concern for patients and members of the public; participants discussed that a technology that is easy to use enables patients to take high-quality images that are better suited for AI analysis and reduces the risk that patients will be unable to use the technology or use it incorrectly. Several members of the public raised the importance of enabling patients to choose how to consult, rather than mandating a consultation type that is inappropriate for them. GPs and NPs’ primary concern was that it should be easy to integrate the AI technology with their current computer and Wi-Fi systems. Dermatologists and patients were concerned about whether the technology would facilitate taking high-quality images for the AI to analyze. AI researchers commented on how practical limitations of a technology can prevent it being used, even if it is potentially very beneficial; therefore, “real-world factors,” such as cost, ease of use, and how well it fits into clinical workflows, need to be considered.

#### Accuracy and Generalizability

Accuracy was raised frequently by all groups and is linked to many of the subthemes. The primary concerns were the false negative rate and the risk of false reassurance and whether the diagnostic accuracy is generalizable to other clinical settings, using different camera technologies, and with other populations and demographics. AI researchers commented that currently AI technologies are often developed on small datasets, which are not representative of the general population and may contain errors and biases and hence, do not generalize well to be accurate in all sections of the population. Members of the public and HCPs were concerned this might mean AI technologies would be less accurate in melanin-rich skin or for rarer skin cancers.

AI researchers discussed how this situation might be improved with close collaboration between clinical and AI researchers and a focus on data quality in a “data-centric” approach. An AI researcher from a commercial background commented on the challenges in collecting a representative dataset for AI development and testing. There are fewer publicly available images of skin lesions in melanin-rich skin, and their app had significantly lower uptake among patients with melanin-rich skin.

#### Evaluation and Regulation

Patients felt that AI technologies should be evaluated by a mix of professionals before they can be adopted, including independent confirmation of diagnostic accuracy. Patients were concerned that AI technology would be adopted based on novelty and hype or because it is cheaper than clinicians’ time when it may not be in the best interests of patients and their clinical care. HCPs felt that significant data from clinical trials would be needed to evaluate AI technologies but recognized that this might take time. AI researchers were concerned that we currently lack good measures to evaluate AI algorithms for use in clinical settings. They stated that the current practice of using simple diagnostic accuracy measures (eg, sensitivity and specificity) is not comprehensive enough to demonstrate the accuracy, benefits, and risks of AI technologies. AI researchers added that, while clinical studies, due diligence, and understanding biases and flaws in an AI system are all important aspects of evaluation, there are other factors, including business sustainability, that need to be considered before a decision on adoption can be made. Many groups commented on the need for health-economic evaluation as part of any evaluation program.

Patients and members of the public often disclosed that they did not understand regulatory processes. Some felt it was important that AI technologies had a Conformité Européene marking and were evaluated by a national body. HCPs were concerned about regulatory processes for AI technologies not being as robust as for medicines and treatments. GPs raised concerns about where the medicolegal responsibility for errors related to the use of AI technologies would lie. Dermatologists wanted the use case for AI technologies to be made clear and for the regulator to assess the technology based on this but acknowledged that regulating AI technologies is challenging. An AI researcher commented that regulatory processes for AI technologies are becoming more complex.

#### Implementation in the UK National Health Service

Members of the public and GPs discussed variation in National Health Service infrastructure in different regions of the United Kingdom, including wireless connectivity, and how this could make implementation difficult.

Most participant groups commented on the capacity of the health care system to implement a new technology where resources are strained. Current pressures may mean that PCPs have insufficient time to understand and implement a new technology. Some HCP participants highlighted that an AI technology with a low specificity might lead to a significant increase in referrals and worsen workload pressures. Conversely, some NPs and dermatologists commented that use of AI technology could help to reduce the number of referrals to secondary care. AI researchers hoped AI technologies could be used alongside PCPs and dermatologists to ease workload pressures.

All groups commented on the importance of professional bodies in the implementation of new technologies. Patients and HCPs felt that implementation would be greatly helped if professional bodies, such as the National Institute for Health and Care Excellence, National Health Service England, Integrated Care Boards, the BAD, or the Medicines and Health products Regulatory Agency, had recommended or evaluated it. GPs and NPs believed any decision to adopt and fund AI technology would need to come from a higher body rather than individual GP practices. AI researchers commented on the importance of the views of professional bodies for the adoption of AI technologies but also on the associated commercial challenges as these bodies vary internationally.

NPs and dermatologists highlighted the need for adequate training in how to use an AI technology as part of the implementation process and clear guidelines on how the AI technology should be used and interpreted.

#### Long-Term Use

The unique potential for AI technologies to continue learning after implementation and for diagnostic performance to improve over time was raised by different participant groups. GPs and patients assumed that this feature would be standard practice and that regular updates would sequentially improve the AI technologies performance as it learned from new data. Few participants commented on how this process could be regulated in practice.

A major concern of all groups was how to check that the AI technology was performing accurately. AI researchers were particularly worried about the potential for the performance of the AI technology to deteriorate over time, referred to as “drift in performance.” They commented that this could occur because of a change in the way the AI technology is used, a change in the population (for example, using the technology in a population with lower skin cancer prevalence or different demographics compared to the development and testing datasets), a change in the hardware, or a change in the accuracy of the technology over time. All groups suggested “sanity checks” that could be used regularly to detect if an AI technology was not performing accurately. These “sanity checks” included expert systems monitoring image metrics over time, comparing the prediction of the new AI technology to the previous AI gold standard; a clinician reviewing all cases that the AI diagnoses as “likely skin cancer” or where AI has low confidence in the diagnosis; or limiting the use of AI technologies with a “human-in-the-loop” approach. AI researchers added that there needs to be an incentive for the makers of the AI technology to maintain and provide ongoing support long-term (Textbox 3).

**Cross-cutting themes.**

**Trust**

- The fact that it hasn’t been the norm within the health care setting, I think will make quite a lot of people feel uncomfortable. So the fact that it’s just so up and coming and new will naturally spark a bit of anxiety in terms of people and they think about if it’s safe, if it’s got the same principles and standards in place in terms of care, safety, confidentiality et cetera. [Patient 1; male, aged <60 years]
- I think there is also a section of the population who are very suspicious of data not being used correctly. So, you would need to have some sort of reassurance about correct data use as well. [Member of the public 4; female, aged >60 years]
- I think people fundamentally put trust in people and we’re still wary about putting trust in machines, or computers, because we’ve been fed sci fi for years that makes us worry, and also I do think that we still think we know best, even though the algorithms that will be in the computer will be absolute gold standard, and they won’t be tired, hung over, jaded, they’ll be right every time. [NP3 (nurse practitioner 3); male, aged <45 years]
- I think the concern at the moment is that there isn’t enough evidence that any of these machines, or machine learning is up to speed to be able to make diagnoses without missing any skin cancer, and that includes rare skin cancers, skin cancers with rare presentations, or in different ethnicities. [Dermatologist 3; female, aged <45 years]
- Yeah. I’m just really unsure in how far, to be honest, machine learning can or will take over a medical setting, or should. The more I see and read, the more I’m getting also suspicious. [AIR3 (AI researcher 3); female, aged <45 years]
- I would question the premise that being a black box is actually a terrible thing or even a novel thing, because clinicians are black boxes. As far as I understand for most medicines we have no idea why they work, and we still use them because statistically it works. And as long as we regularly check that our models work statistically then who cares whether that’s a chemical or a computational black box. Obviously, it’s nicer if you can also present the clinician with some data that the clinician then can use to make further judgment calls. [AIR1; male, aged <45 years]
- As a field we’re only beginning to scratch the surface of what that means, to trust technology. If say my physician tells me, this is a good way to understand more about your skin conditions, there’s some element where I trust he or she as a professional and the information relayed is therefore accurate, and therefore that trust extends to the thing they had suggested. [AIR5; male, aged <45 years]

**Ease of use and acceptability**

- So, being accurate, being honest, easy to use, easy to understand. And easy for the GPs to use as well, the other health care professionals who’ve got to get the information through that as well, easy for them. Because they’re going to have to look at whatever it’s come up with and try and make some sense of that before they actually sit down in front of us. [Member of the public 1; female, aged >60 years]
- For me patient choice is so important—so both have a machine in the GP surgery for those who would prefer to do that and have an app for the people who are quite comfortable using those. [Member of the public 1; female, aged >60 years]
- I suppose if it was a lengthy process. So if there was dodgy Wi-Fi or it’s hard to get your image or it’s taking time to upload...I suppose it’s just the practicalities and the ease of the software and hardware that you’re using, those will be barriers, if it didn’t work. [NP1; female, aged >45 years]
- How easy is it to take the photograph? I think that’s really important. Because I get loads of referrals with photographs, but the photographs are completely useless, they’re blurred and a complete waste of time. Somebody’s ticked the box and said, sent a photograph, but they might as well not be there. [Dermatologist 1; female, aged >45 years]
- If you have a technology that’s extremely beneficial for a certain disease but it’s really expensive, it takes a really long time, and it’s hard to use then no one will ever use it. So there’s real world factors there. [AIR5; male, aged <45 years]

**Accuracy and generalizability**

- I think the key issue is generalisability, because algorithms are developed on a very small set which have very specific properties and there is biases. And then of course, they do not work on a wider range of other images from other scanners from other countries...this is a huge issue, the generalisability. You have all these algorithms that achieve higher numbers in one setting but it doesn’t mean that they will work well on new data they have not seen. [AIR3; female, aged <45 years]
- They need to make sure that there is a proper diversity of people in there. Because AI is only ever going to be as good as what you use to train it. So I think they need to be particularly careful about diversity of skin color and capturing the wide variety that’s needed. [Member of the public 13; female, aged >60 years]
- For us it’s a kind of a chicken and egg problem. So, there’s not a lot of data for darker skin available, so training on that...and especially proving the accuracy on dark skin it’s almost impossible...We don’t have the users, we don’t get the data, we don’t get the proof of how good we are on darker skin—so that definitely is a loop that we need to break at some point. [AIR4; male, aged >45 years]

**Evaluation and regulation**

- I have come across many issues because algorithms have been evaluated with measures that are actually not measuring what you would want or need in a clinical setting...So I actually think the most important thing right now would be to set up proper evaluation schemes and to think about how deep learning models should be or can be properly evaluated...The real question is, how do we evaluate them to make sure they will work in a medical clinical setting. [AIR3; female, aged <45 years]
- So this is definitely a concern, not to send too many users into healthcare. The first concern’s accuracy, we don’t want to miss too many skin cancers, for sure, but the second concern is definitely also the health economic case. So, we can of course send a huge amount of people into healthcare. We find more skin cancer, we reach our goals of finding more skin cancer, that’s fine. But we don’t solve a real problem, we make it worse for the healthcare system as well. [AIR4; male, aged >45 years]
- I worry a little bit that if it’s something that is a manageable cost it might be overused. It’s a way to shift a number of patients who you might otherwise see...They’re always on an efficiency drive, they’re always under pressure to save money...I don’t know whether, if the technology was affordable for GP surgeries, they would just think, oh great...we’re short of doctors we can just process people through the hands-off routine...you stop thinking about it in clinical terms and you start thinking about it in financial terms. [Patient 2; female, aged <60 years]
- I think that’s really important that it has CE marking; because I see a lot of apps and crap in the digital space that a lot of people are buying and spending money on and they’re not evidence-based medicine. [Member of the public 2; female, aged <60 years]
- I know it (regulation) is fairly patchwork. Obviously, when it comes to medication and prescribing, there’s fairly robust regulatory systems... When it comes to certain equipment, some patient devices it’s a bit piecemeal. But I would be fairly reassured if the MHRA covered this equipment. [GP3; male, aged <45 years]
- I’ve been indoctrinated by the British Association of Dermatologists. And what they explained. They gave a position statement, I think it was a year or two ago, and we were’ve told these are medical devices and therefore they need to go through these medical regulatory agencies. I think before Brexit it obviously would have been Europe as well and now I think it’s MHRA should be responsible for this. Which makes sense because obviously it’s a very important thing for patients and for doctors, medicolegally as well. So it needs to work for its purpose, for what they say it’s going to be doing. [Dermatologist 3; female, aged <45 years]
- And also where does it stand legally? If you come to me and you show me a mole and I do my AI thing and I say to you computer says no, and off you go and you keep doing the things that you do, and then two years later you come back to me with a large black blobule, sentinal lymph node biopsy positive...is that my fault? Is that the AI’s fault? And if it’s the AI’s fault, who are you suing? [GP6; female, aged >45 years]

**Implementation in the UK National Health Service (NHS)**

- I think when you’re an external looking in at the NHS it’s just a monster beast to try and understand different parts of it...Overall I think integration of digitals is great, but it’s not uniform, and also there are massive connectivity issues still in some areas...Some parts of the country are doing amazing things, and other parts are so backward. [Member of the public 2; female, aged <60 years]
- I’m just very much aware that the basics of general practice is under so much pressure that adding a new technology and complication is not everybody’s first priority...I appreciate a lot of these new technologies can save time and resources in the long term, but certainly, if I’m thinking now of the start of a difficult winter, it’s going to be a tough sell right now. [GP3; male, aged <45 years]
- One of the things I came across is that there was a shortage of dermatologists in general. This means that the practitioner is expected to see a lot of patients in the same day...the practitioner is a human, maybe some stress, or he had a bad day, he might not spot some feature in that image. So those (AI) tools can be something that fixes this gap if in that day he had a bad day and didn’t notice some features. [AIR2; male, aged <45 years]
- So it’s going to cost something to have this kind of level of equipment, but there’s not an infinite amount of money in GP land, if they’re spending on that then it’s coming out of somewhere else, which might mean we won’t have the money or equipment for something that may benefit patients. [NP2; female, aged <45 years]
- It’s got to come with the support and the training along with it, it’s not just about getting a new piece of kit...I think the important thing is that that cost incorporates training and a clear understanding of the device and what it’s used for, and why, and how to use it. It’s silly to give a piece of kit and then not have the support to know how to use it best. [NP1; female, aged >45 years]

**Long-term use**

- In some ways the accuracy would build as the technology was used. So the longer it’s been in play the more you can depend on it probably...I assume that you don’t just test the heck out of it and say it’s fine now and then stop refining it. [Patient 2; female, aged <60 years]
- Let’s say the model was trained in a way it works really well when you are taking dermatoscope photos, and it gives you a really good sense of the malignancy potential there and the studies support this. Then a patient sends you a photo...and this photo was taken using a smart phone with different kinds of lighting conditions, in a setting where it was actually never designed for use but that fact is not obvious to anyone who hasn’t been thinking about this for a really long time. So with the best of intentions, I think you could still have a situation where the performance starts drifting away from how it was designed for use. [AIR5; male, aged <45 years]
- Maybe scanners will change...It is not clear that if an algorithm works well on the scanner of the last generation, even from the same company, (if) you get a new one that it would work on that as well...They (scanners) will not stay the same, but then we really should think about how do we integrate new modalities into the machine learning models, because it’s not sustainable to develop something on a fixed dataset. [AIR3; female, aged <45 years]
- It should be tested before it gets to the GP surgery, and then it should be checked...at regular intervals, to make sure it’s still working correctly. Because if you start falsely diagnosing people, that could be a complete waste of time. [Patient 3; female, aged >60 years]
- If we look at a top-level view across all AI it’s really hard to summarise in a single sentence whether this will help or hurt. But if developed well, if used well I still do believe there’s lots of great potential here. [AIR5; male, aged <45 years]

## Discussion

### Principal Findings

In this study, many of the discussions centered on 2 simple questions: who is going to use the AI technology, and what is it going to do? Most participants commented on positioning AI technology with patients or allied HCPs (before the PCP) or with the PCP as a decision support tool. Few participants proposed positioning the technology to triage referrals to secondary care or as a decision aid for dermatologists. Participants highlighted several overarching topics as important to them, including trust, acceptability, generalizability, evaluation, regulation, implementation, and long-term use.

The risk of false negatives resulting from an AI technology with poor sensitivity and missing skin cancers was a major concern for all participant groups. Missed skin cancers, especially melanoma, are likely to lead to late diagnosis and worse outcomes for patients [3,6]. One potential benefit of AI technology mentioned by all groups was the reduction in the workload of health care services, primarily through effective triage of patients that need to see PCPs or specialist clinicians. This could be the case if the AI technology has a good specificity with few false positive results. False positives are inevitable, but an AI technology with a low specificity could significantly increase the workload of health care services. This was a concern raised by HCPs, but few other participant groups commented on this risk. It has been suggested that overdiagnosis of melanoma is rising due to increased rates of skin examination, decreased thresholds for biopsying skin lesions, and for labeling morphological changes on histopathological examination as malignant [25]. Implementation of an AI technology with low specificity could increase both rates of skin examination and biopsy rates, both potentially contributing to overdiagnosis.

AI researchers were the most pragmatic, expressing concerns about the generalizability of many AI technologies and the relevance of current testing approaches in preparation for clinical implementation. They were concerned about the robustness of the datasets underlying AI technologies, including the representativeness of skin cancer prevalence and patient demographics in the datasets. They felt that, at the current time, these technologies need to be implemented with a human in the loop. Many HCPs were aware of the lack of evidence and potential risk to patients that has been publicly commented on by the BAD [9]. Patients and the public were aware of potential improvements in patient access, diagnostic accuracy, and reduced workload AI technologies could offer but were concerned about the risk of missing cancers and losing the opportunity for human interaction and to ask questions.

Complexity underpins most of the generated themes. Developing, evaluating, implementing, regulating, and maintaining AI technology in health care settings are all multifaceted, complex processes containing many opportunities for error, as laid out in the CanTest framework [14]. Therefore, it is unsurprising that so many diagnostic technologies fail at the implementation stage [26]. Several studies have discussed the complexity of implementing digital, AI, and machine learning (ML) interventions into health care settings; they recommended a whole-of-system approach with particular focus on how users interact with devices and user training [12,27].

### Comparison With Existing Literature

The NASSS framework [15] was chosen to guide this study because it includes a wide range of domains that help capture the complexity of implementing health care interventions [28]. Most of the cross-cutting themes link closely to NASSS domains, specifically “knowledge to use the technology,” “demand-side value (to patient),” “adopters,” and “organizations.” There were several themes raised that did not fit into the NASSS framework (such as the potential for continued learning with AI technologies), as well as themes (such as trust and acceptability) that seemed to lose some of their breadth and nuance by being contained within the NASSS domains. We chose to code both inductively and deductively, which allowed us to better capture participants’ views and build knowledge about applying the NASSS framework to novel AI technologies.

A recent Swedish study used an AI-based melanoma decision support aid for clinicians as an example technology to generate discussions with participants about the implementation of decision aids. In keeping with their findings, many participants in our study discussed the issues of accuracy, safety, data security, liability, ease of use, and integration [29]. Our findings support those of a recent systematic review on the use of ML-based risk prediction models in health care settings [13] in which participants demonstrated largely positive views of AI technologies but identified many barriers. Echoing findings from several recent studies [30-33] we identified concerns about diagnostic accuracy, risk of patient harm, ease and speed of use, HCP overreliance on the technology, legal liability, data protections, data quality, impersonality, and positioning in the diagnostic pathway. In particular, we identified aspects of the consultation participants felt that AI technologies could not replicate, that is, the need for human interaction and clinical experience and judgment. Participants commented on the risk that AI technologies will not be effective in minority populations who are inadequately represented in training and testing datasets and may exclude populations with lower technological literacy, such as older people.

The wide variety in positioning and approach of existing AI technologies [7,8] indicates that the optimal position and approach have yet to be determined. Several participant groups highlighted that they wanted more evidence of the accuracy of technologies in real-life clinical use. This fits with recent reviews of AI technologies aimed at detecting skin cancer [7,8,33], although increasing evidence from clinical trials is emerging [10]. PCPs were keen to have an AI technology to support their diagnostic decision-making, in keeping with findings from a previous study [34]. AI researchers highlighted a growing body of research in AI technology development for health care settings, including the “human-in-the-loop” approach and the “data-centric AI movement” [12,35,36].

Regulation was a topic raised by several participant groups. The fast-moving pace of AI development makes regulation of AI technologies challenging: underregulation risks patient safety while overly zealous regulatory approaches could hinder AI development pipelines and implementation [37,38]. AI has the unique potential to continue developing over time as it is exposed to more data. Regulatory bodies around the world are attempting to keep up with the rapid developments in AI technologies. In the United States, the Food and Drug Administration has proposed the 510k pathway, which facilitates the approval of software as a medical device if it is substantially based on a previously approved technology [39], and is developing an approach to an AI-ML workflow that would enable continued learning after implementation [40]. There are emerging national and international regulatory policies, including the European AI Act, the United States AI Bill of Rights, and the United Kingdom policy on AI [41-43].

### Strengths and Limitations

To the authors’ knowledge, this is the first study to report the views of a broad range of stakeholders about the use of AI technologies to facilitate the earlier diagnosis of skin cancer in primary care settings. We had good variation among interviewees in terms of background, age, sex, and geographical location. The study benefited from PPI at every stage, and a strong conceptual framework was used to develop the framing of the interview schedule and data coding.

Aiming to recruit a wide range of stakeholders was a conscious choice, as we felt this was important to achieve a breadth of opinions; the trade-off was that time and resources meant we were only able to include limited numbers in each participant group. The aim was to achieve breadth of opinion without necessarily achieving saturation. AI and clinical implementation are complex subjects, which meant that we were more likely to recruit participants with higher educational attainment and who are engaged with health care research or AI; both these aspects may have affected the balance of views we obtained. We only recruited 1 participant who reported that they disapproved of using AI technologies to help diagnose skin cancer. Skin cancer is more common in melanin-poor skin; however, a key limitation of current AI technologies is their lack of training and testing in populations with melanin-rich skin. We recruited limited numbers of participants with melanin-rich skin in this study, so participant views on this issue may be incomplete. In contrast to other participants, AI researchers were largely based outside the United Kingdom, reflecting the location of the majority of commercial companies developing these technologies. However, it meant their knowledge of United Kingdom clinical practice and diagnostic pathways was sometimes limited.

### Implications for Clinical Practice, Research, Adoption, and Policy

Health care services are working under extreme pressures in primary and secondary care [44]. AI technologies aimed at diagnosis or triage of skin lesions could facilitate early diagnosis of skin cancer to improve outcomes for patients and potentially ease some of these pressures. However, before this can happen, research is required to prove their efficacy with real-world clinical populations and to address the questions that remain about the most effective positioning of the technologies in the diagnostic pathway and the optimal approach for their use. Diagnostic technologies that are used in populations that are different from those they were developed and tested in are prone to spectrum bias [45]. Better measures of clinical performance are required to inform these studies, which consider not only diagnostic accuracy but also provide a measure of generalizability and dataset quality [46].

Some of our findings can be used to further develop the NASSS framework, for example, to consider in more depth how users interact with an AI technology and the potential for continued development after implementation. When developing an AI technology aimed at the diagnosis or triage of skin cancer, developers need to consider carefully and be specific about the intended use, including where it will fit into the diagnostic pathway for skin cancer and the approach that it is going to take. Developers must also consider the quality and representativeness of the data they use to develop the AI.

The decision to adopt an AI technology is complex and multifaceted. Clear regulatory processes that consider unique features of AI technologies need to be established, including continued learning, to ensure AI technologies are safe and effective when used in clinical settings. Adopters should also consider what safety nets are in place to identify poor performance and reduce false negative results, such as expert systems and regular “sense checks.”

### Conclusions

AI technologies are being designed with a wide variety of approaches, and the optimal approach and position in the skin cancer diagnostic pathway for these technologies have not yet been determined. AI technologies have the potential to help detect and diagnose skin cancer, to improve patient experience and outcomes, and to reduce the workload of overstretched health care systems. However, we have identified important concerns surrounding trust, acceptability, usability, generalizability, evaluation, regulation, implementation, and long-term use. These technologies need to be developed carefully and thoughtfully to achieve their potential, guided by evidence-based approaches and appropriate implementation, taking into consideration long-term sustainability and safety.

## Appendix

Multimedia Appendix 1 Interview schedule for patients and members of the public.

Multimedia Appendix 2 Interview schedule for health care professionals and artificial intelligence researchers.

Multimedia Appendix 3 COREQ (Consolidated Criteria for Reporting Qualitative Research) checklist.

## Abbreviations

AI: artificial intelligence
BAD: British Association of Dermatologists
COREQ: Consolidated Criteria for Reporting Qualitative Research
GP: general practitioner
HCP: health care professional
KC: keratinocyte cancer
ML: machine learning
NASSS: Nonadoption, Abandonment, and Challenges to Scale-Up, Spread, and Sustainability
NP: nurse practitioner
PCP: primary care practitioner
PPI: Patient and Public Involvement
