# Supplementary material for: User and Developer Views on Using AI Technologies to Facilitate the Early Detection of Skin Cancers in Primary Care Settings: Qualitative Semistructured Interview Study
Source: JMIR Cancer. 2025 Jan 28;11:e60653. doi: 10.2196/60653 (PMC11815299; doi:10.2196/60653)
Supplement: Multimedia Appendix 1 [file cancer_v11i1e60653_app1.doc]

**
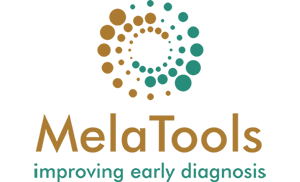
**

**The use of Artificial Intelligence/Machine Learning (AI/ML) technologies to help your family doctor/general practitioner (GP) spot skin cancer earlier**

**Part 1: Views on using Artificial Intelligence Technologies to help with the early diagnosis of skin cancer by family doctors/GPs**

**Initial open questions**

1. What does the term artificial intelligence mean to you? What about machine learning?
2. How do you think AI is used in healthcare at the moment?
3. How do you think that AI technologies could help with diagnosing skin cancer?

**Further questions and prompts for exploring the issues raised:**

1. **How the technology would be used**

- Where should the AI technology be available? (For example, should it be given to patients to use at home, to GPs or nurses to use at your local doctor’s surgery, or in separate clinics that the local doctors or nurses can refer patients to, in hospital clinics, or a combination of these options?).
- What design of AI technology do you think would work best if it were used in local doctor’s surgeries? (e.g., specially designed diagnostic device, or photos taken using camera/smartphone to be uploaded to computer)? And how long do you think the test should take?
- How would you like to see this technology being used to help make a decision about moles? (e.g., to help decide whether the mole needs to be referred to a skin specialist, to provide a specific diagnosis, or another method)

1. **What do you think are the most important features that the technology should have?**

- How accurate do you think AI technologies need to be? Is the accuracy more important for melanoma (the most dangerous skin cancer), or is accuracy for other skin cancers and non-malignant moles equally important?
- Do you have any views on what would be a reasonable cost for these technologies?
- How long would it be reasonable for the test to take?
- What feedback should the AI technologies give? (e.g. traffic light risk score of red/amber/green to show the level of worry about the mole, a single diagnosis, list of what it thinks are the 3 most likely diagnoses, a number to show how confidence the AI is in its diagnosis)?

1. **What do you think would be the effect if these technologies were to be brought in?**

- What do you see as the benefits of using AI technologies to assess moles?
- What do you see as the harms of using AI technologies to assess moles?
- What could help these technologies to be used in local doctors’ surgeries?
- What could stop these technologies from being used in local doctors’ surgeries?
- How do you think using AI technologies could affect the NHS? (e.g., demand on GPs and hospital skin specialists, waiting times to see GPs and hospital specialists, and how easy it is for patients to be seen)
- What impact do you think these technologies could have on the number of patients referred to hospital specialists (e.g., over investigation and over diagnosis, missed diagnoses)?
- What impact do you think that using AI technologies could have on doctors’ skills and confidence in diagnosing skin cancers and other skin conditions?
- Do you think there are other ways that using AI technologies could affect the diagnosis of skin conditions (other than doctors’ skills and confidence as mentioned in the last question)?

1. **How do you think these types of technologies should be assessed and regulated?**

- Who do you think should test and assess AI technologies? (e.g., the technology developers, NHS, university researchers)
- How do you think AI technologies should be assessed? (E.g., is a formal trial in clinics needed? Do they need to be tested in local doctors surgeries?)
- Who do you think should advise that AI technologies are safe and effective enough to be used with patients? (For example, the NHS, the government, the doctors and nurses using the technologies, or someone else?)

**Part 2: Impact of Covid-19 on participant’s views on using AI/ML technologies in medicine**

1. During the Covid-19 pandemic lots of technologies have started to be used in medicine, such as video consulting, text messages, and email communication.
   - Has this changed your views on the use of technology in medicine/healthcare?
   - Has this changed your views on the use of tests in healthcare (FIT as an example)?
   - And has this affected your views on using AI technologies in medicine/healthcare?
2. Do you think that increased use of technology, including video consultations, is a good thing? Or a bad thing? Which do you prefer face-to-face consultations, telephone consultations, or video consultations? Or do you have mixed/other views on it?
