# Supplementary material for: User and Developer Views on Using AI Technologies to Facilitate the Early Detection of Skin Cancers in Primary Care Settings: Qualitative Semistructured Interview Study
Source: JMIR Cancer. 2025 Jan 28;11:e60653. doi: 10.2196/60653 (PMC11815299; doi:10.2196/60653)
Supplement: Multimedia Appendix 2 [file cancer_v11i1e60653_app2.doc]

**
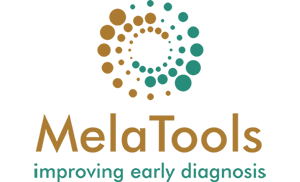
**

**The use of Artificial Intelligence/Machine Learning (AI/ML) technologies to aid the early diagnosis of skin cancer in primary care settings**

**Part 1: Views on the use of AI/ML technologies to aid with the early diagnosis of skin cancer in primary care settings**

**Initial open questions**

1. If I were to say artificial intelligence and machine learning (AI/ML), what do the terms mean to you? (Clinicians only)
2. How or when do you think AI/ML is used in healthcare at the moment?
3. Have you ever used AI/ML in your practice? (Clinicians only)
4. How do you think that AI/ML technologies could help with diagnosing skin cancer?

**Further questions and prompts to explore issues raised:**

1. **How do you think AI/ML technologies could best be used in clinics?**

- Where in should the technology be available? (e.g., given to patients, in nurse clinics, GP clinics, specialist diagnostic clinics (such as the rapid access diagnostic clinics), secondary care, or a combination of these)?
- What design of AI/ML technology would work best in primary care (e.g., specially designed diagnostic device, or images taken using camera/smartphone to be uploaded to computer)?
- How would you like to see this technology being used in the assessment of skin lesions? (e.g., to triage skin lesions that need referral or biopsy, to provide a specific diagnosis, or another method)

1. **What do you think are the most important features for the AI/ML technology to have?**

- How accurate do you think AI/ML technologies need to be? Is the accuracy more important for melanoma, or is accuracy for other skin cancers and benign lesions equally important?
- Do you have any views on what would be a reasonable cost for these technologies?
- How long would it be reasonable for the test to take?
- What would you like to see as the output from these technologies (e.g., traffic light risk assessment, single diagnosis, differential diagnosis, estimates of confidence in the diagnosis)?

1. **What do you think would be the effect if these technologies were to be implemented?**

- What do you see as the benefits of delivering care through AI/ML technologies?
- What do you see as the harms of delivering care through AI/ML technologies?
- What are the facilitators to implementing AI/ML technologies in primary care?
- What are the barriers to implementing AI/ML technologies in primary care?
- How do you think the introduction of AI/ML technology would affect the NHS? (e.g., demand on GPs and hospital skin specialists, waiting times to see GPs and dermatologists, and how easy it is for patients to be seen)
- What impact do you think these technologies could have on referral rates (e.g., over investigation and over diagnosis, missed diagnoses)?
- What impact do you think that using AI/ML technologies could have on doctors’ skills and confidence in diagnosing skin cancers and other skin conditions?
- Are there other ways that using AI/ML technologies could affect the diagnosis of skin conditions (other than doctors’ skills and confidence as mentioned in the last question)?

1. **How do you think these types of technologies should be evaluated and regulated?**
2. Who should test and evaluate AI/ML technologies?
3. How should they be evaluated? (e.g., is a clinical trial needed? Do they need to be validated in primary care settings?)
4. Who should advise that AI/ML technologies are safe and effective enough to be used with patients? (E.g., NHS England, NHS digital, MHRA, government, the clinicians using the technologies, or someone else?)

**Part 2: Impact of Covid-19 on the participant’s views on the use of AI/ML technologies in medicine**

1. During the Covid-19 pandemic there has been increased use of technology in medicine, for example video consulting, text messages, and email communications.
   - Has this changed your perspective on the use of technologies in medicine/healthcare?
   - And has this affected your views on the use of AI/ML technologies in medicine/healthcare?
   - Has this changed your views on the use of diagnostic tests in healthcare (e.g. FIT)?
2. What are your views on the increase in automation and virtual/remote consulting? Is it a positive thing? Do you prefer face-to-face consultations? Or do you have mixed/other views on it?
