# Supplementary material for: User and Developer Views on Using AI Technologies to Facilitate the Early Detection of Skin Cancers in Primary Care Settings: Qualitative Semistructured Interview Study
Source: JMIR Cancer. 2025 Jan 28;11:e60653. doi: 10.2196/60653 (PMC11815299; doi:10.2196/60653)
Supplement: Multimedia Appendix 3 [file cancer_v11i1e60653_app3.pdf]

Consolidated criteria for reporting qualitative studies (COREQ): 32-item checklist

No. Item	Guide questions/description	Reported on Page #
**Domain 1: Research team and reflexivity**		
Personal Characteristics		
1. Interviewer/facilitator	Which author/s conducted the interview or focus group?	6
2. Credentials	What were the researcher's credentials? E.g. PhD, MD	1,6
3. Occupation	What was their occupation at the time of the study?	6
4. Gender	Was the researcher male or female?	Not reported
5. Experience and training	What experience or training did the researcher have?	6
Relationship with participants		
6. Relationship established	Was a relationship established prior to study commencement?	Not reported as not applicable
7. Participant knowledge of the interviewer	What did the participants know about the researcher? e.g. personal goals, reasons for doing the research	Not reported as not applicable
8. Interviewer characteristics	What characteristics were reported about the inter viewer/facilitator? e.g. Bias, assumptions, reasons and interests in the research topic	Not reported as not applicable

**Domain 2: study design**		
Theoretical framework		
9. Methodological orientation and Theory	What methodological orientation was stated to underpin the study? e.g. grounded theory, discourse analysis, ethnography, phenomenology, content analysis	Page 7
Participant selection		
10. Sampling	How were participants selected? e.g. purposive, convenience, consecutive, snowball	Page 5, 6, and 8
11. Method of approach	How were participants approached? e.g. face-to-face, telephone, mail, email	Page 5
12. Sample size	How many participants were in the study?	Page 8
13. Non-participation	How many people refused to participate or dropped out? Reasons?	Not applicable, reasons reported on page 8
Setting		
14. Setting of data collection	Where was the data collected? e.g. home, clinic, workplace	Page 6
15. Presence of non-participants	Was anyone else present besides the participants and researchers?	Not reported as not applicable (participants were invited to bring a family member or friend with them, but no participants opted to do this)
16. Description of sample	What are the important characteristics of the sample? e.g. demographic data, date	Page 8. Date of interviews on page 6
Data collection		
17. Interview guide	Were questions, prompts, guides provided by the authors? Was it pilot tested?	Page 6
18. Repeat interviews	Were repeat inter views carried out? If yes, how many?	Not reported as not applicable
19. Audio/visual recording	Did the research use audio or visual recording to collect the data?	Page 6
20. Field notes	Were field notes made during and/or after the inter view or focus group?	Page 6/7
21. Duration	What was the duration of the inter views or focus group?	Not reported

22. Data saturation	Was data saturation discussed?	Yes but we moved away from this concept, see page 6/7
23. Transcripts returned	Were transcripts returned to participants for comment and/or correction?	Not reported as not applicable
**Domain 3: analysis and findings**		
Data analysis		
24. Number of data coders	How many data coders coded the data?	Page 7
25. Description of the coding tree	Did authors provide a description of the coding tree?	Page 7
26. Derivation of themes	Were themes identified in advance or derived from the data?	Page 7
27. Software	What software, if applicable, was used to manage the data?	Page 7
28. Participant checking	Did participants provide feedback on the findings?	Not reported as not applicable
Reporting		
29. Quotations presented	Were participant quotations presented to illustrate the themes/findings? Was each quotation identified? e.g. participant number	Pages 12, 13, 14,15, 18, 19, and 20
30. Data and findings consistent	Was there consistency between the data presented and the findings?	See results section pages 9-20
31. Clarity of major themes	Were major themes clearly presented in the findings?	See results section pages 9-15
32. Clarity of minor themes	Is there a description of diverse cases or discussion of minor themes?	See results section pages 15-20 and discussion section pages 21-25
